# Functions of nonsuicidal self-injury in a Hungarian community adolescent sample: a psychometric investigation

**DOI:** 10.1186/s12888-021-03613-4

**Published:** 2021-12-09

**Authors:** Melinda Reinhardt, Gyöngyi Kökönyei, Kenneth G. Rice, Boglárka Drubina, Róbert Urbán

**Affiliations:** 1 Department of Personality and Health Psychology, Institute of Psychology, Faculty of Education and Psychology, ELTE Eötvös Loránd University, Budapest, Hungary; 2 Child and Adolescent Psychiatry, 14th District Medical Center, Budapest, Hungary; 3 SE-NAP2 Genetic Brain Imaging Migraine Research Group, Hungarian Academy of Sciences, Semmelweis University, Budapest, Hungary; 4 Department of Pharmacodynamics, Faculty of Pharmacy, Semmelweis University, Budapest, Hungary; 5 Center for the Study of Stress, Trauma, and Resilience, Department of Counseling and Psychological Services, Georgia State University, Atlanta, USA; 6 Doctoral School of Psychology, ELTE Eötvös Loránd University, Budapest, Hungary

**Keywords:** Nonsuicidal self-injury, Inventory of Statements About  Self-Injury, Confirmatory factor analysis, Exploratory structural equation modeling, Gender invariance, Adolescents

## Abstract

**Background:**

The Inventory of Statements About Self-Injury (ISAS) is a psychometrically valid tool to evaluate the motives of nonsuicidal self-injury (NSSI), but there are a few studies that test gender differences in the factor structure of the measurement. However, several differences across gender were identified in NSSI (e.g., in prevalence, methods, functions). Therefore, our study focused on further analyses of the dimensionality of the ISAS functions.

**Methods:**

Among Hungarian adolescents with a history of NSSI (*N* = 418; 70.6% girls; mean age was 16.86, SD = 1.45), confirmatory factor analysis and exploratory structural equation modeling frameworks were used to test the factor structure of the ISAS part II.

**Results:**

Results support the two-factor structure of the questionnaire. Intrapersonal and interpersonal motivation factors emerged in the whole sample, but this factor structure varied across gender. Among girls, intrapersonal motivation of NSSI was associated with higher loneliness, more inflexible emotion regulation, and a more pronounced level of internalizing and externalizing mental illness symptoms.

**Conclusions:**

Our findings provide sufficiently solid arguments for the need to examine NSSI functionality separately for adolescent girls and boys because there were clear gender differences in the motives underlying NSSI. In addition, precise scanning of patterns of NSSI functions may further help us to identify the most at-risk adolescents regarding self-injury.

**Supplementary Information:**

The online version contains supplementary material available at 10.1186/s12888-021-03613-4.

## Epidemiology of nonsuicidal self-injury in adolescence

Nonsuicidal self-injury (NSSI) comprises deliberate and conscious self-injurious acts without the intention to die. These acts can cause immediate physical damage to the body tissue, and include behaviors such as cutting, scratching, biting, burning, and hitting oneself [1].

These socially unacceptable behaviors are the most common during adolescence [2]. According to different surveys, the lifetime prevalence of NSSI ranges between 7.5 and 46.5% in community adolescent samples [e.g., 3, 4, 5]. A systematic review of more than 50 studies concluded that the mean lifetime prevalence of NSSI behaviors was 18% during adolescence [6]. However, this review drew attention to a substantial difference in the estimates of lifetime prevalence of NSSI depending on the method of assessment. While a single binary item assessment showed a 12.5% average lifetime prevalence, multiple items or behavior checklist methods indicated almost twice (23.6%) the lifetime prevalence [6].

In Hungary, for the most part, two NSSI measurements have been used in research. According to the Deliberate Self-Harm Inventory (DSHI; [7]), a 17-item questionnaire that assesses type(s), frequency, severity, and duration of NSSI, 17.1% of a representative Hungarian adolescent sample had engaged in NSSI, which was the lowest rate among 11 European countries [6]. However, in 2020, 23.6% of nonclinical and 53% of clinical Hungarian adolescents reported NSSI based on the DSHI [8]. Using the Self-Injury Questionnaire – Treatment Related (SIQ-TR; [9]), 34.4% of Hungarian justice-involved juveniles had a history of NSSI before the past month, and 26.3% reported current self-injurious behavior within the past month [10]. In this vulnerable adolescent population, the lifetime prevalence of NSSI was very high (60.7%) and quite similar to Hungarian [8] and Dutch (66.4%; [11]) results for clinical youth.

Research has consistently shown that adolescent girls are at greater risk of NSSI than boys, especially in clinical populations [12]. There is also a gender difference in the most common methods of NSSI based on a community adolescent sample; girls mainly engage in self-cutting and carving their skin, whereas boys hit themselves [13]. Another important confounding factor would be cultural differences in NSSI prevalence [3] and the functionality of NSSI [14]. Moreover, using multiple forms of NSSI is more likely to be linked to more severe intrapersonal and interpersonal problems [15] and probably to more dysfunctional emotion regulation.

## Motivation for engaging in NSSI

The Four Function Model (FFM; [16]) is one of the most cited functionality models of NSSI. According to the FFM theory, NSSI acts can serve as intrapersonal (“automatic”) together with interpersonal (“social”) mechanisms, and both processes can reinforce the behavior positively or negatively. In this model, self-injury can (1) decrease negative emotional experiences (automatic-negative reinforcement) or (2) generate a desirable positive emotional state (automatic-positive reinforcement), and can (3) reduce (social-negative reinforcement) or (4) induce specific interpersonal experiences (social-positive reinforcement). Automatic (intrapersonal) functions are much more common than social (interpersonal) functions [17].

Another comprehensive framework of NSSI functionality, the two-function model, emphasizes intrapersonal and interpersonal functions of NSSI [18]. Nock and Prinstein [16] also identified the more parsimonious two-factor solution, what they referred to as automatic and social NSSI motives. However, for theoretical reasons, Nock and Prinstein [16] argued for the four-function framework. However, most of the subsequent studies could not support the four-function solution [e.g., 18, 19]. Furthermore, there have been several statistical anomalies associated with this structure [18].

In a review, Klonsky [20] affirmed that affect-regulation is the leading motive of engaging in NSSI. Self-punishment was also a common reason for NSSI. Still, there was less evidence of anti-dissociation (e.g., causing pain to try to feel something), anti-suicide (e.g., avoiding the impulse to attempt suicide), sensation-seeking (e.g., entertaining someone by doing something extreme), as well as interpersonal-influence (e.g., seeking care from others) and interpersonal boundaries (e.g., creating a boundary between the self and others) functions of NSSI [20].

These results were also confirmed in a meta-analysis, which included 53 independent samples [21]. Intrapersonal functions of self-injury were more prevalent (66-81% of participants) than interpersonal motives (32-56% of participants). In particular, the aim of avoiding or escaping from an unwanted internal state was the most common intrapersonal function. In contrast, self-punishment and inducing positive feelings via self-injury were the less frequent motives. Communicating distress was the most frequent interpersonal function, whereas punishing or hurting others was the least frequent [21]. However, Jarvi et al.’s [22] systematic review pointed out that social contagion (i.e., influence of social media and friends) plays a strong role in predicting the first engagement in NSSI. In constrast, repeated NSSI is mainly influenced by intrapersonal functions [22]. In a large adolescent and adult clinical population, Victor et al. [23] did not find any differences between females and males in interpersonal functions of NSSI. They also noted that males listed significantly lower levels of intrapersonal functions than females.

However, gender differences have been detected in deliberate self-cutting: the rate by which female adolescents state they cut themselves because they want to punish themselves is twice that of males [24]. A similar gender pattern emerged for the reason of reducing an unwanted state of mind [24]. Whitlock et al. [25] showed that female college students more likely engaged in NSSI because they were upset or would have liked to attract attention. Males more likely indicated they engaged in NSSI because of anger and stated that intoxication was a starting point of NSSI acts [25]. Nonetheless, a study, which involved seven countries, did not present gender and cultural differences in NSSI motives, finding instead that girls reported more reasons behind self-injury than boys, and that older girls more frequently used self-injury as a cry for help act than younger ones [26].

## Analysis of NSSI-functions based on the Inventory of Statements About Self-Injury

The second part of the Inventory of Statements About Self-Injury (ISAS Part II; [27]) was developed to assess the underlying causes of NSSI. In the initial study, exploratory factor analysis (EFA) results indicated that the set of 13 empirically substantiated motives can be classified into two broader, intrapersonal and interpersonal functions. These two robust factors harmonized with Nock and Prinstein’s FFM model [16]. The intrapersonal factor was theoretically comparable with the automatic factor, and the interpersonal factor was comparable to the social factor [27]. Klonsky and Glenn [27] concluded that the two-factor structure was not affected by gender and ethnicity, however they did not indicate whether measurement invariance was tested.

The intrapersonal factor comprised five subscales: affect-regulation, anti-dissociation, anti-suicide, marking distress, and self-punishment. The interpersonal factor comprised eight subscales: autonomy, interpersonal boundaries, interpersonal influence, peer-bonding, revenge, self-care, sensation seeking, and demonstrating toughness [27]. Only in one of the 13 functionality scales emerged some uncertainty: factor loadings of self-care were marginally different in the case of the interpersonal (.41) and intrapersonal (.33) factors. Furthermore, Klonsky and Glenn [27] considered that self-care (when someone engages in self-injury to create a physical wound to care about instead of experiencing emotional distress) would be a better conceptual fit for the intrapersonal factor.

The two larger factors showed excellent internal consistency, and had reasonable correlations with clinical phenomena such as mood and borderline personality disorder symptoms, and suicidality [27]. Due to its psychometric support, the use of the ISAS has quickly spread in NSSI studies.

Indeed, subsequent studies strengthened support for the two-factor framework of the ISAS Part II. Based on EFA results, in 2015, Klonsky and his colleagues affirmed the intra-, and interpersonal factors in a large clinical sample with a wide age range (from 11 to 73 years old) [18]. Consistent with earlier research, they found some ambiguity with the self-care subscale in which one item loaded on the intrapersonal factor while the other two items loaded on the interpersonal factor [18].

Among others, in English-speaking countries (e.g., UK, USA, Canada, Australia) Kortge et al. [28] also supported the intra-, and interpersonal function factor structure of the ISAS with EFA. In this research, the self-care subscale clearly loaded onto the intrapersonal factor. In this way, the intrapersonal factor comprised six subscales, while the interpersonal factor brought together seven subscales. Based on Rasch analysis, Kortge and her colleagues demonstrated that the most relevant functions for the respondents were interpersonal boundaries and toughness, and peer bonding was the least relevant function. As regards the intrapersonal factor, the strongest endorsed functions were affect-regulation and self-punishment whereas the weakest was the motive of self-care. Item functioning was independent of age, nationality, and education level [28].

Among Turkish high school students [29], and in a Spanish clinical sample [30], confirmatory factor analysis (CFA) supported the two-factor model. Bildik et al. [29] found that self-care loaded onto the interpersonal factor, whereas Pérez et al. [30] found that it loaded on the intrapersonal factor. However, in the Turkish study, the correlation was particularly high between the two factors, supporting a general function factor with specific NSSI motives [29].

As a result of the relatively low subsample size of patients with borderline personality disorder, Pérez et al. [30] acknowledged they could not test invariance based on diagnosis. Importantly, to our knowledge, none of the prior studies of the ISAS Part II have examined gender or age invariance in the factor structure.

Intrapersonal and interpersonal motivations of NSSI may relate to mental health problems and psychosocial functioning in different ways, although the picture is not entirely clear. Klonsky and Glenn [27] found that, compared to effect involving interpersonal functions, intrapersonal motives were more strongly linked to depressive symptoms, borderline personality symptoms, and suicidal ideation. However, there were no substantial differences between the two superordinate motive factors in associations with anxiety symptoms and history of suicide attempts. It should be highlighted that rumination could be a moderator between depression and endorsement of intrapersonal functions of NSSI [31]. In addition, Nock and Prinstein [32] reported a significant relationship between interpersonal motives and depressive symptoms, and between interpersonal motives and loneliness.

## Current study

Based on the review of previous studies on the ISAS, our first aim was to validate the questionnaire and provide NSSI demographics in a Hungarian community adolescent sample. We also aimed to analyze the factor structure of the ISAS functions (ISAS Part II short version). In this context, our purpose was to further discuss the dimensionality of the ISAS functions, both in a confirmatory factor analysis (CFA) and exploratory structural equation modeling (ESEM) framework. Beyond the traditional CFA, ESEM analysis can provide a more realistic test of the theoretical constructs and a more accurate estimation of factor intercorrelations. Moreover, no study to our knowledge has evaluated the short form of the ISAS Part II using ESEM. Shorter scales are more practical tools for large sample studies as well as for clinical settings, therefore testing the factor sturcture of the short form of the ISAS Part II was a relevant aim of the current study.

In addition, due to the lack of testing measurement invariance of the ISAS functions in the previous research, we examined measurement invariance across gender. Testing gender differences in the dimensionality of the ISAS Part II was also justified because several dissimilarities (e.g., in prevalence, methods, and underlying motives) across gender have been described in NSSI [12, 13, 23].

In order to examine concurrent validity, a further objective was to explore the associations between the functionality factors and relevant variables. To that end, we examined associations with externalizing and internalizing mental health symptoms, certain emotion regulation aspects (self-critical rumination and experiential avoidance), and methods of the NSSI behavior. Our research could provide further understanding of the associations between NSSI functions and different psychological reactions to stressors (i.e., mental health problems and special maladaptive emotion regulation strategies). We hypothesized that internalizing mental health problems and dysfunctional emotion regulation would be more strongly linked to intrapersonal functionality of NSSI than to interpersonal motives. Furthermore, we assumed that intrapersonal functions play a more important role in engaging in most NSSI methods than interpersonal motives.

## Method

### Participants and procedure

The cross-sectional study conducted between February 2019 and January 2020, and involved 14 secondary schools seated in the capital and rural towns throughout of Hungary. One class at each school within each grade level (9-12) was selected at random to participate. Thus, the sample represented all grades across the secondary schools. Participants completed a questionnaire package in the classroom or computer room under the supervision of trained principal investigators. No teaching staff were present. Based on the technical possibilities of the schools, the completion of self-report questionnaires was carried out online on computers or mobile devices using the Qualtrics platform or on paper.

We invited 1232 students to participate, however, 173 students were absent during data collection or declined to participate. On the whole, 1059 students participated in the study. Following a review of missing data, 44 respondents were excluded from the data analysis. These participants stopped before completing the penultimate (SDQ; [33]) and the last questionnaires (ISAS; [27]). Thus, the final sample contained 1015 adolescents. All participants were Hungarian.

Participants’ ages ranged from 14 to 20 years (age M = 16.81 years, SD = 1.42). Females represented 66.1% of the sample and males represented 33.7%; two students (0.2%) failed to respond to this question. Major analyses were performed on the 418 adolescents (41.2% of the entire sample) who reported having engaged in NSSI. Of that group, 70.6% were female (*n* = 295) and 29.2% were male (*n* = 122); one did not provide gender data (0.2%).

Female (M = 16.80; SD = 1.40) and male (M = 17.01; SD = 1.52) respondents who engaged in NSSI were similar in age, *t*(411) = 1.31, *p* = 0.192, d = 0.15. Based on urban and rural classification, there was also not a gender difference in residence, χ^2^(2, *N* = 416) = 0.97; *p* = 0.616; φ = 0.05); that is, girls resided in the capital (25.9%) and countryside (74.1%) nearly the same ratio as boys (28.7 and 71.3%, respectively).

Participation in the study was voluntary and anonymous. All students and one of their parents gave written informed consent to participate in the study. The research plan was approved by the ELTE Eötvös Loránd University Faculty of Education and Psychology Research Ethics Committee, and the study was carried out in accordance with the Declaration of Helsinki [34].

### Measures

#### Nonsuicidal self-injury

We measured NSSI with the Inventory of Statements about Self-Injury (ISAS) part I and II [27]. The Hungarian version of the ISAS was administered, which was developed with the agreement of the original author, E. D. Klonsky.

The first section of the questionnaire detects the lifetime frequency of 12 different self-injurious behaviors performed intentionally and without suicidal intent (e.g., cutting, biting, severe scratching, banging or hitting self). Five questions associated with other characteristics of NSSI are also addressed in part I (date of first self-injurious act; experienced physical pain during NSSI; was self-injurer alone when he/she performed self-injury and has he/she wanted to stop self-injury; how much time elapses between the urgency and the act of self-injury).

The second part of the inventory includes 13 functions of NSSI. In the original version, each function is assessed by three items on a 3-point scale, ranging from 0 to 2 (0 = *Not relevant*, 1 = *Somewhat relevant*, 2 = *Very relevant*). Several studies divided these motives into intrapersonal and interpersonal function categories [e.g., 27, 30, 35]. Intrapersonal functions include affect regulation, anti-dissociation, anti-suicide, marking distress, and self-punishment. Interpersonal functions refer to autonomy, interpersonal boundaries, interpersonal influence, peer-bonding, revenge, self-care, sensation seeking, and toughness [27]. Recall that in some studies, self-care related to the intrapersonal factor [28, 30].

Good reliability coefficients were obtained for both intrapersonal (α = .80), and interpersonal factors (α = .88) [27]. Washburn et al. [36] assessed each function by the two most representative items on the same 3-point scale as in the original study. Accordingly, in the short version of the second part of the ISAS, sum scores for each of the 13 functions can range from 0 to 4. We used this short version of the ISAS Part II in the current study to reduce participation demands on the adolescents and schools. Cronbach alphas were similar to the results of Klonksy and Glenn [27] in our study; α = .76 for the intrapersonal, and α = .82 for the interpersonal factor.

We also included one question from the Self-Injury Questionnaire – Treatment Related (SIQ-TR; [9]) to assess NSSI more specifically. Respondents provide information about the frequency (i.e., severity) of the act(s) based on the number of days the given NSSI behavior occurred during the last month.

#### Mental health screening

Mental health problems, both externalizing and internalizing symptoms, were assessed with the self-report form of the Strengths and Difficulties Questionnaire (SDQ; [33]). The instrument has 25 items allotted into five subscales: Emotional symptoms, Conduct problems, Hyperactivity/inattention, Peer relationship problems, and Prosocial behavior. A total difficulties score can be computed based on the first four factors. The Internalizing symptom subscale reflects Emotional symptoms and Peer relationship problems, while Externalizing symptom subscale integrates Conduct problems and Hyperactivity/inattention items. Participants were asked to score the items on a scale from 0 to 2 (0 = *Not true*, 1 = *Somewhat true*, 2 = *Certainly true*). Higher scores on the first four subscales (symptomatic scales) indicate more severe problems. In contrast, on the Prosocial behavior subscale, higher ratings refer to more prosocial activities.

In this study, the internal consistency of the total symptoms scale and the Internalizing symptom subscale as measured by Cronbach alpha was adequate (α = .75, and α = .75), while Externalizing symptom and Prosocial behavior subscales provided satisfactory internal consistency for research purposes (α = .64, and .67, respectively). These internal consistency scores compare well with the original questionnaire [33]. Our analyses only used the total symptom, the Internalizing and Externalizing symptom, and the Prosocial behavior subscales.

#### Emotion regulation

##### Self-critical rumination

Self-critical perseverative style of thinking was assessed by the 10-item Self-Critical Rumination Scale (SCRS; [37]). Items are rated on a 4-point scale (from 1 = *Not at all* to 4 = *Very much*). The single-factor structure questionnaire showed excellent internal consistency in the original study (α = .92; [37]), as well as in our current study (α = .91).

##### Experiential avoidance

Avoidance and Fusion Questionnaire for Youth (AFQ-Y8; [38]) is an 8-item scale that measures tendencies of experiential avoidance and cognitive fusion as markers of psychological inflexibility. Items are rated on a 5-point scale from 0 = *Not at all true* to 4 = *Very true*. The factor structure of the AFQ-Y8 reflects one factor, with higher scores showing stronger experiential avoidance. The scale had good internal consistency in the initial study (α = .83 in [38]). In this current study, Cronbach alpha was acceptable (α = .70).

#### Loneliness

A single item was used to measure feelings of loneliness (“Do you feel lonely?”). Respondents rated the item using a 4-point scale (1 = *Never*; 2 = *Sometimes*; 3 = *Often*; 4 = *Very often*).

### Data analysis

To understand the factor structure of motives of NSSI, we parcelled two items of each motives resulting in 13 observed indicator variables scored from 0 to 4. We used these observed variables as ordinal indicators because we observed severe floor or ceiling effects in the vast majority of motives.

We tested measurement models using the more restrictive CFA approach, which fixes all cross loadings in zero, and more flexible exploratory structural equation modeling (ESEM) approach, which allows cross-loadings which may better represent the complexity of NSSI [cf., 39]. Furthermore, the flexible ESEM approach also allows to the use of statistical advances of structural equation modeling, including goodness of fit statistics, inclusion of correlated uniquenesses, multiple indicators multiple causes models (MIMIC models), and tests of multiple group invariance [40].

Confirmatory factor analyses and exploratory structural equation modeling (ESEM) were performed with M*plus* 8.0 [41]. The analyses were based on Weighted Least Squares Mean and Variance adjusted (WLSMV) estimation [42, 43]. Missing values were treated with full maximum likelihood function implemented in M*plus*.

The first step was to test a one-factor, overall NSSI motives model. The second step was to test a two-factor model of intrapersonal and interpersonal NSSI functions described in the work of Klonsky and Glenn [27]. Modification indices detected substantial cross-loadings, so we also tested the two-factor ESEM model with target rotation which allows cross-loadings. Although we also planned to investigate the measurement invariance across gender, the inspection of factor loadings revealed large differences in the pattern of associations across gender. Therefore, we concluded that at this stage configural gender invariance could not be supported. As a result, we did not continue the formal tests of measurement invariance.

In the next step, we investigated the multiple indicators multiple causes (MIMIC) models [42, 44] to estimate the effects of covariates such as experiential avoidance, self-critical rumination, and loneliness. Furthermore, we also evaluated the associations between NSSI motives and internalizing and externalizing symptoms, along with prosocial behavior. We estimated the two sets of covariates separately because of the medium and large correlations among covariates may cause multicollinearity problems in the model. As the final step, factor scores were calculated for further analyses, including multinomial logistic regression analysis to predict the type(s) of NSSI reported.

In the CFA and ESEM analyses, the satisfactory degree of fit requires the comparative fit index (CFI) to be larger than 0.90 and preferably 0.95 or larger, root mean square error approximation (RMSEA) below 0.05 (excellent fit) or between 0.05 and 0.08 (adequate fit), and standardized root mean square residual (SRMR) below 0.10.

## Results

### Descriptive statistics of NSSI behaviors

A total of 418 adolescents in the total sample (41.2%) reported a history of NSSI, and of those, which 76.8% (*N* = 321, 31.6% of the whole sample) reported current NSSI behavior within the past month. Most of the adolescents who engaged in current self-injury (88.5%; *N* = 284) did so from 1 to 5 days in the past month, another 3.7% (*N* = 12) reported self-injury between 6 and 10 days, 2.2% (*N* = 7) between 11 and 15 days, and 5.6% (*N* = 18) more than 15 days.

There were gender differences in the lifetime prevalence of self-injury, χ^2^(1, *N* = 1013) = 6.43, *p* = 0.011, φ = 0.08). Adolescent girls tended to engage in self-injury at a higher rate (44.0%; *N* = 295) than boys (35.7%; *N* = 122); however, the effect size was small. There was also a significant difference between girls (79.7%; *N* = 235) and boys (70.5%; *N* = 86) in the rate of current (past month) prevalence of self-injury, χ^2^(1, *N* = 417) = 4.10, *p* = 0.043, φ = 0.10).

Among those who engaged in NSSI, the most common types of behaviors were banging or hitting self (53.1%; *N* = 222) and interfering with wound healing (52.2%; *N* = 218). Cutting (40.7%; *N* = 170), biting (39%; *N* = 163), pinching (38.8%; *N* = 162), and severe scratching (34.4%; *N* = 144) were also relatively frequent. Swallowing dangerous substances was the less prevalent method for self-injury (7.2%; *N* = 30). The frequencies of different types of NSSI behavior according to occasional (< 10) and recurrent (≥10 lifetime episodes) self-injury are presented in Table 1. Compared to boys, girls reported significantly higher frequencies of using three types of self-injury such as cutting, χ^2^ (1, *N* = 417) = 18.69, *p* < .0001, φ = 0.21), carving, χ^2^ (1, *N* = 417) = 6.57, *p* < .01, φ = 0.13), and severe scratching, χ^2^ (1, N = 417) = 16.36, *p* < .0001, φ = 0.20). On the contrary, boys reported higher frequencies of banging or hitting self, χ^2^ (1, N = 417) = 4.98, *p* < .026, φ = 0.11).

**Table 1:** Frequencies of nonsuicidal self-injury among participants who engaged in self-injury

	Less than 10 times	10 times or more
Types of self-injury	n (%)	n (%)
Cutting	98 (23.6)	72 (17.3)
Biting	76 (18.2)	87 (20.8)
Burning	51 (12.3)	27 (6.5)
Carving	82 (19.7)	34 (8.2)
Pinching	66 (15.8)	96 (23.0)
Pulling hair	37 (8.9)	41 (9.8)
Severe scratching	56 (13.4)	88 (21.2)
Banging or hitting self	87 (20.8)	135 (32.4)
Interfering with wound healing	60 (14.5)	158 (38.1)
Rubbing skin against rough surface	34 (8.2)	39 (9.4)
Sticking self with needles	49 (11.7)	57 (13.6)
Swallowing dangerous substances	23 (5.5)	7 (1.7)
Other	9 (2.2)	21 (5.0)

Note. *N =* 414-417

The average age when respondents engaged in self-injury at the first time was 11.97 years (SD = 3.55), with the highest prevalence between the ages of 12 and 15. In our study, we did not seek to examine whether who engaged in NSSI satisfy the criteria of nonsuicidal self-injury disorder (NSSID; DSM-5;[45]). However, we considered it important to define repetitive and occasional NSSI. Based on Gratz et al. [46], almost two thirds of those who engaged in self-injury in the present sample (68.7%; *N* = 287) had a history of recurrent NSSI (≥10 lifetime episodes of NSSI), while 31.3% (*N* = 131) could be described as who occasionally engaged in self-injury (< 10 lifetime episodes of any type(s) of NSSI). Adolescents who engaged in self-injury applied 3.94 NSSI methods on average (SD = 2.59; range between 1 and 11 methods). No gender difference was found in the frequency (repetitive vs. occasional) of the self-injury episodes, χ^2^(1, *N* = 394) = 1.58, *p* = 0.21, φ = 0.06), as well as in versatility of NSSI (i.e., number of NSSI methods, χ^2^(11, *N* = 417) = 19.05, *p* = 0.06, φ = 0.21), however, the effect size was large in the latter case.

Thirty-three percent (33.8%; *N* = 129) of those who engaged in self-injury experienced pain during the act, 41.1% (*N* = 157) sometimes experienced pain, while 25.1% (*N* = 96) reported no pain. The majority of those who engaged in self-injury were alone during the act (62.2%; *N* = 237), 22.8% (*N* = 87) indicated they were sometimes alone, while 15% (*N* = 57) were not alone at that time. Forty-two percent (42.2%; *N* = 154) engaged in NSSI in less than an hour when they experienced the urge to self-injury, while 44.4% (*N* = 162) were able to wait more than a day. Most of the participants who engaged in self-injury would like to discontinue engaging in NSSI (82.3%; *N* = 302). However, 17.7% (*N* = 65) had never wanted to stop NSSI behaviors.

Girls were more likely to engage in NSSI because of affect regulation (t[349] = 3.32, *p* < 0.001, Cohen’s d = 0.51), self-punishment (t[349] = 3.48, p < 0.001, d = 0.42), and anti-dissociation (t[349] = 3.20, *p* = 0.002, d = 0.38) motives. At the same time, boys were more likely to engage in NSSI because of sensation seeking (t[349] = 3.37, p < 0.001, d = 0.41; see Supplementary Table 1).

### Dimensionality of NSSI motives

We performed several CFAs to test several alternative models of NSSI motives. The fit indices of tested models are reported in Table 2. First, we tested the one-factor model which yielded unacceptable fit. We also tested the two-factor model based on a previous study by Klonsky and Glenn [27]. This model also yielded unacceptable fit. In order to understand the source of misfit, modification indices were inspected and we found indications for several cross-loadings. Instead of allowing cross-loadings in the CFA, we applied exploratory structural equation modeling (ESEM). This approach allowed us to keep the two theoretical factors and cross-loadings which may responsible the local misfit.

**Table 2:** Fit indices of alternative measurement models

	χ^2^	df	CFI	RMSEA [90%CI]	SRMR
One-factor model	621.5	65	0.689	0.157 [0.146-0.168]	0.127
Two-factor model (Klonsky & Glenn, 2009)	489.9	64	0.762	0.138 [0.127-0.150]	0.111
Two-factor ESEM model (Klonsky & Glenn, 2009)	175.2	53	0.932	0.081 [0.068-0.095]	0.052
Two-factor ESEM model (Klonsky & Glenn, 2009) with modification*	134.3	52	0.954	0.067 [0.053-0.081]	0.045
Gender difference					
Boys	89.1	52	0.960	0.086 [0.054-0.116]	0.059
Girls	102.2	52	0.954	0.062 [0.044-0.080]	0.049

*: Error correlation is allowed between Toughness and Sensation seeking. *N =* 349

The two-factor ESEM model yielded acceptable fit. After the inspection of modification indices, we allowed the error correlation between toughness and sensation seeking, which seemed plausible given their content; further analyses were based on this modified measurement model. Factor loadings of this model are presented in Table 3. In the total sample of participants who engaged in self-injury, we observed large cross-loadings of marking distress, interpersonal boundaries, toughness, and autonomy. This analysis supported the two-factor model, but also revealed that some motives are complex because they have significant loadings on both factors such as marking distress, interpersonal boundaries, toughness and autonomy. Furthermore, results indicated that the anti-suicide items fit better as indicators of the interpersonal factor rather than as indicators of the intrapersonal factor.

**Table 3:** Factor Loadings from Exploratory Structural Equation Modeling

	Total		Boys		Girls	
	Intrapersonal	Interpersonal	Intrapersonal*	Interpersonal*	Intrapersonal	Interpersonal
Affect regulation	**0.78**	0.13	−0.10	**0.57**	**0.81**	0.16
Self-punishment	**0.75**	0.16	0.02	**0.61**	**0.77**	0.16
Anti-dissociation	**0.81**	0.25	−0.07	**0.74**	**0.83**	0.24
Anti-suicide	0.03	**0.54**	**0.58**	**0.50**	0.00	**0.53**
Marking distress	**0.69**	**0.52**	0.16	**0.90**	**0.69**	**0.49**
Interpersonal boundaries	**0.52**	**0.53**	**0.34**	**0.70**	**0.58**	**0.53**
Self-care	0.13	**0.73**	**0.67**	**0.72**	0.13	**0.69**
Sensation seeking	0.27	**0.48**	**0.37**	**0.70**	**0.33**	**0.38**
Peer bonding	−0.18	**0.77**	**1.00**	**0.57**	−0.20	**0.71**
Interpersonal influence	0.15	**0.70**	**0.52**	**0.74**	0.11	**0.71**
Toughness	**0.41**	**0.45**	−0.12	**0.53**	**0.44**	**0.55**
Autonomy	**0.30**	**0.78**	**0.49**	**0.74**	**0.33**	**0.82**
Revenge	0.24	**0.70**	**0.67**	**0.79**	0.30	**0.61**
Factor correlations	−0.25		−0.34		−0.33	

Rotation is a target rotation. Salient loadings (> 0.30) are boldfaced. *N =* 348. *N*_*boys*_ *=* 96. *N*_*girls*_ *=* 252. *: the original factor name is used

In further analyses, we performed the ESEM in boys and girls separately. Inspecting the factor loadings implied that different factor structure migth be present in both genders, indicating the problem with configural invariance itself. While the pattern of significant factor loadings in girls reflects the theoretical model, this is not the case in boys. In this latter group, all motives loaded significantly on one factor, and a few motives loaded on a separate ‘peer-bonding’ factor. However, we also performed the traditional measurement invariance testing. Due to the ordinal nature of indicators with WLSMV estimator and M*plus* defaults, the configural models was compared with the scalar invariance model. The model implying equal loadings and equal thresholds yielded significantly worse model fit compared to the model without those equality constraints, Δχ^2^ (59, *N* = 349) = 84.0, *p* = .0179. However, due to the small sample size of boys we did not investigate this difference further, and restricted our analyses to the girls’ sample.

In the girls’ sample, the original two-factor model was supported with intrapersonal and interpersonal motive factors. The variables with the highest loadings on intrapersonal factors were affect-regulation, self-punishment and anti-dissociation, and importantly these variables did not have salient loadings on the interpersonal factor. The variables with the highest loadings on interpersonal factors and negligible cross-loadings on the other factor were peer bonding, interpersonal influence, self-care, anti-suicide and revenge. Several items loaded saliently on both factors such as marking distress, interpersonal boundaries, sensation seeking, toughness and autonomy. These motives may have both intra- and interpersonal components.

### Construct validity of NSSI motives: concurrent predictive validity of NSSI motives

We tested whether intrapersonal and interpersonal factors are associated with any types of self-injury among girls. For the multinomial regression analysis, we recoded the lifetime frequencies of using each type of self-injury into three categories: no use as a reference category, low frequency of use (less than 10 times) and high frequency of use (10 or more times of use). The factor scores of the main (intrapersonal and interpersonal) motives were used as explanatory variables, and age was controlled in all regression models (Table 4).

**Table 4:** Concurrent Predictive Validity of the Two Latent NSSI Motives: Multinomial Regression Analyses Among Girls

	Less than 10 times		10 times or more		
Type of self-injury	Intrapersonal motives^#^	Interpersonal motives^#^	Intrapersonal motives^#^	Interpersonal motives^#^	*R*^*2*^
Cutting	***2.01*** [1.32-3.06]***	1.40 [0.97-2.02]	***8.37*** [4.54-15.44]***	1.51 [0.91-2.50]	30.5%
Biting	*1.83* [1.21-2.76]**	1.02 [0.67-1.54]	***3.01*** [1.89-4.79]***	1.47 [0.94-2.32]	13.2%
Burning	*2.11* [1.29-3.44]**	1.02 [0.62-1.68]	***3.99*** [1.96-8.11]***	1.45 [0.74-2.84]	13.2%
Carving	1.29 [0.88-1.90]	1.34 [0.92-1.95]	***2.88*** [1.62-5.11]***	1.15 [0.64-2.06]	9.9%
Pinching	1.18 [0.76-1.83]	0.87 [0.56-1.35]	***1.99*** [1.35-2.93]***	0.82 [0.54-1.23]	10.1%
Pulling hair	*1.96* [1.13-3.29]*	0.87 [0.49-1.54]	*2.59* [1.47-4.58]**	0.81 [0.44-1.48]	10.7%
Severe scratching	***2.45*** [1.50-4.00]***	1.14 [0.72-1.80]	***3.35*** [2.18-5.16]***	1.07 [0.71-1.60]	20.3%
Banging or hitting self	1.38 [0.92-2.06]	0.82 [0.55-1.23]	***2.33*** [1.58-3.44]***	1.22 [0.83-1.79]	10.0%
Interfering with wound healing	0.86 [0.55-1.35]	0.81 [0.51-1.29]	1.14 [0.82-1.60]	1.19 [0.84-1.69]	6.3%
Rubbing skin against rough surface	***3.55*** [1.81-6.97]***	*2.40* [1.27-4.56]**	*1.98* [1.17-3.36]*	1.23 [0.72-2.12]	13.4%
Sticking self with needles	1.31 [0.83-2.05]	0.94 [0.59-1.51]	*2.01* [1.26-3.21]**	0.90 [0.59-1.59]	5.7%
Swallowing dangerous substances	***4.79*** [2.00-11.49]***	1.07 [0.44-2.60]	*5.65* [1.23-26.01]*	3.21 [0.74-13.90]	21.9%

Odds ratio [95% Confidence interval]. ^#^: Motives were based on factor scores. Age was controlled. R^2^ is Nagelkerke R.^2^ Italicized values are significant at *p* < 0.05. Based on a Bonferroni correction, bold and italicized values are significant at *p* < 0.002. Reference group is 0 (when the specific type of self-injury is not reported). *N =* 251

High frequency of use was predicted only by the intrapersonal motive factor except for interfering with wound healing, however, the odds ratios varied from 8.37 (cutting) to 1.98 (rubbing skin against rough surface). The low frequency of use of each type of NSSI was also predicted by the intrapersonal motive factor with exception of carving, pinching, banging or hitting self, interfering with wound healing, and sticking self with needles. The interpersonal motive factor predicted only the low frequency of rubbing skin against rough surface (Table 4).

### Explanatory variables of NSSI motives: multiple indicators-multiple causes

We also tested in one model if experiential avoidance and self-critical rumination as transdiagnostic symptoms explain any variance of NSSI motives among girls. We also included here loneliness and age as covariates. Standardized regression coefficients are presented in Table 5. Loneliness, experiential avoidance, and self-critical rumination predicted intrapersonal motives significantly. So higher loneliness, higher experiential avoidance, and stronger self-critical rumination were associated with higher intrapersonal motives. The interpersonal motive factor was explained by age and loneliness. Younger age and lower loneliness were associated with stronger interpersonal motive.

**Table 5:** Explanatory Variables of NSSI Motives Among Girls

Model	Intrapersonal motives	Interpersonal motives
Model 1		
Age	0.11	−0.16*
Loneliness	0.27***	− 0.22*
Experiential avoidance (AFQ-Y8)	0.24**	0.03
Self-critical rumination (SCRS)	0.20*	−0.11
*R*^*2*^	36.6%	10.2%
Model 2		
Internalizing symptoms (SDQ)	0.40***	−0.20**
Externalizing symptoms (SDQ)	0.15*	0.14
Prosocial behavior (SDQ)	0.03	0.05
Age	0.09	−0.17*
*R*^*2*^	21.6%	7.6%

AFQ-Y8 = Avoidance and Fusion Questionnaire for Youth. SCRS=Self-Critical Rumination Scale. SDQ = Strength and Difficulties Questionnaire. *N* = 251

We tested in another model whether internalizing and externalizing symptoms, and prosocial behavior are linked with NSSI motives. Both internalizing and externalizing symptoms were linked with intrapersonal motives. So higher internalizing and externalizing symptoms associated with higher intrapersonal motives however, the link seems to be stronger in case of internalizing symptoms. Internalizing symptoms and age, on the other hand, negatively associated with interpersonal motives, thus higher score on internalizing symptoms and older age is associated with lower interpersonal motives. Correlations for the variables are summarized in Supplementary Table 2.

## Discussion

Several current studies reported growing incidence of NSSI in nonclinical adolescent populations [4]. However, only a few valid, reliable, and complex NSSI measurements exist. Furthermore, testing of psychometric properties of existing questionnaires is also scarce. Our aim was therefore twofold: firstly, to present extensive self-injury demographics in a large Hungarian adolescent sample, and secondly, to further test the factor structure of the ISAS part II [27] taking into account possible gender differences. To the best of our knowledge, this is the first study that has used an ESEM approach in testing the functionality factors of the ISAS part II.

In our sample, 41% of the secondary school pupils had already engaged in some form(s) of NSSI. This relatively high lifetime prevalence rate is parallel to the ceilings of self-injury measurements in community adolescent samples [3, 4]. In this respect, it should be recalled that only one-third of the adolescents who engaged in any NSSI acts do so less than 10 times in their life. This group comprised teens who engaged in self-injury occasionally [46], whereas the other two-thirds engaged in NSSI repetitively. Future research should clarify precisely the possible differences between occasional and repetitive self-injury in NSSI functions and other related variables (e.g., mental health aspects, emotion regulation, psychosocial functioning).

Compared to a recent Hungarian survey of self-injury [8], in our study, we detected 17% more lifetime prevalence of NSSI. One of the reasons for this discrepancy maybe the difference between the two self-injury measurement used. Horváth et al. [8] defined the frequency of NSSI episodes as whether the respondent answered “yes” to any of the listed self-injurious behaviors. However, in our study, participants estimated the number of times they had intentionally performed the listed types of NSSI. In the latter case, higher prevalence of NSSI might be more likely to occur because respondents have to more thoroughly consider their engagement in self-injury.

Nearly one third of our nonclinical adolescent sample reported current self-injury. It is worth to noting that the majority of those engaging in self-injury reported doing so from 1 to 5 occasions in the previous month. Only 3.6% of the total sample indicated more frequent NSSI in the past month. This subgroup is particularly vulnerable regarding repetitive self-injury. These results are also consistent with former studies which revealed NSSID prevalence between 1.5 and 6.7% in nonclinical adolescent samples [47]. Such findings demonstrate the need to introduce effective and multiple behavior checklist methods into secondary prevention of NSSI among youth. The ISAS could be a low cost but comprehensive tool in order to screen the frequency, forms, and motives of self-injury in school environments to prevent further NSSI-associated physical and psychological health issues (e.g., functional impairment; [48]).

On this point, another crucial aspect is the age of target population. Similar to previous studies [49, 50], the onset of self-injury, on average, in our sample typically occurred at the age of 12, emphasizing the need for NSSI prevention awareness in lower secondary schools.

More than half of those who engaged in self-injury used hitting and/or interfering with wound healing. Cutting, biting, pinching and severe scratching were also common. Swallowing dangerous substances was the least frequent method, which could be linked to its invasive and more drastic nature. Moreover, swallowing poisonous substances is more strongly associated with suicide attempts than NSSI. According to the World Health Organization, self-poisoning with pesticide accounts for 20% of suicide worldwide [51].

Completely in line with former studies [12], adolescent girls reported higher lifetime and point prevalence of NSSI in our survey. However, it is important to underline that there was no gender difference in two indicators of severity (frequency and versatility) of NSSI. Future studies might examine the possible background of these results at different age levels in adolescence. In our sample, girls engaged in cutting, carving, and severe scratching in greater proportion than boys, whereas boys had higher rates of hitting self. These results are similar with former studies among nonclinical adolescents, where different forms of scraping the skin were more common among girls, but hitting self was more typically used by boys [13].

Regarding the factor structure of the ISAS part II, our results based on ESEM analysis strenghten support for the two-factor structure of NSSI motives. In addition, results added interpretive clarity to the two function factors. These results are very similar to previous findings [18, 27–30, 52]. In our analysis, affect regulation, self-punishment and anti-dissociation functions purely loaded onto the first factor which can be described as an intrapersonal motive factor. However, anti-suicide function, which traditionally belongs to intrapersonal motives, loaded strongly on the interpersonal factor. Therefore, in this study, the second factor which can be desribed as an interpersonal motive factor is made up self-care, sensation seeking, peer-bonding, interpersonal influence, revenge, autonomy, and anti-suicide self-injury motives.

An important emergent question based on these results would be how the anti-suicide function of NSSI could be related to interpersonal processes. A conceivable explanation would be that, in adolescence, suicidal thoughts are often linked with imagined reactions of family and friends to a possible fatal outcome. Moreover, it is also common that young people are concerned about the impact of their possible death on their immediate environment. In this context, adolescents easily attribute relational and communicative value to suicidal thoughts or behaviors. Alternatively, they might endorse such thoughts or engage in NSSI behaviors to avoid the impulse to attempt suicide. Furthermore, suicide and suicidal thoughts are socially stigmatized phenomena [53], therefore interpersonal attitudes could be linked to this category.

Our results can strenghten former analyses which identified self-care as an interpersonal NSSI motive [e.g., 27, 29]. Nonetheless, marking distress, interpersonal boundaries, and demonstrating toughness motives may have both intra- and interpersonal components, whereas these possible functions of NSSI loaded on both factors at nearly the same strength.

Furthermore, these motives showed large cross-loadings. Each of these three functions may be interpreted as processes which can regulate the boundaries between the self and not-self. Therefore, the question arises whether certain motives of self-injury could reflect to difficulties in distinguishing the internal (self) and external boundaries (reality, relationships). Flexibly separating the self from others is a main developmental task in adolescence. However, as Erikson [54] observed, becoming more autonomous, working out individual identity in a complex way, and managing relationships can increase self-uncertainity. In further studies, it would be interesting to examine possible links between certain NSSI functions and adolescent self-concept, including aspects of emotional maturity.

The most relevant functions of the intrapersonal factor were anti-dissociation and affect-regulation. This is parallel with former results which identified affect regulation as a leading motive in NSSI [20]. For the interpersonal factor, autonomy and peer-bonding were the most relevant functions. These results can be well-integrated into the two-function model of NSSI [18], as well as in the two-factor solution (automatic and social) from Nock and Prinstein’s FFM model [16]. Intrapersonal aspects of NSSI can help with managing intolerable emotional experiences (automatic reinforcement), while interpersonal facets can assist in attaching to or detaching from others (social reinforcement).

Contrary to findings reported by Kortge and colleagues [28], peer bonding was a strongly endorsed motive for the interpersonal factor. This can be explained by considering findings that pointed to a significant peer socialization effect of friends mainly on younger adolescents’ NSSI behavior [55]. This process can be linked to the effects of social media that have been heightened over the past decade. A systematic review concluded that online social networking can lead to increasing involvement of NSSI behavior as young people share their thoughts and practices about NSSI with each other or seek social support from their companions through social media platforms [56].

In our study, the intrapersonal and interpersonal factors correlated weakly, possibly partly because the individual indicators were allowed to cross-load. However, this result and other findings indicate that the two overall factors are considerable separate motivations behind NSSI.

We found that the factor structure of the ISAS part II was different between for girls and boys. Therefore, we could not analyze metric and scalar gender invariance. Among girls, the factor structure was identical with the analysis in the whole sample. At the same time, among boys all of the NSSI motives loaded onto the interpersonal factor with the addition that eight motives had simultaneous loadings on both the inter- and intrapersonal factors. These sets of factor loadings reflect differences in the NSSI construct for boys compared to girls, and suggest several related speculations. Perhaps in certain motives (e.g., anti-suicide, self-care, peer-bonding, revenge) social processes could help to channel intrapersonal motivations for boys. For example, for boys, engaging in NSSI because of self-care could be an indicator to alert the social milieu to provide some care (care from others) but at the same time, NSSI may operate as a tool by which boys trigger positive emotions (care from the self).

Furthermore, the marking distress function had a salient loading on the interpersonal factor among male adolescents. These results reflect the possibility that boys interpreted the use of self-injurious acts as a cry for help communication addressed to their environment. In addition, due to the small sample size of adolescent boys, we limited subsequent validity and explanatory analyses to data based on the girls.

Among adolescent girls, we found evidence for concurrent predictive validity of NSSI functions. The intrapersonal motive factor explained a high frequency (10 or more times) of use of almost all the NSSI methods (except interfering with wound healing). Cutting, swallowing dangerous substances, and burning were predicted the strongest by intrapersonal motives of self-injury. Our results are consistent with former studies which demonstrated that intrapersonal functions are much more frequently behind NSSI than interpersonal functions [17]. Coping with negative emotions, self-punisment, and reducing dissociation as motivations can potentially increase the frequency of NSSI. In particular, NSSI among women and in clinical samples is frequently linked to affect-regulation (i.e., cutting; [57]).

Furthermore, we were able to demonstrate among girls that possible transdiagnostic factors such as self-critical rumination and experiential avoidance [58], as well as loneliness explained more than one third of the variance in intrapersonal functionality. Higher use of specific inflexible emotion regulation strategies and loneliness have a great impact on the emergence of intrapersonal motivations for engaging in self-injury.

Additionally, higher levels of internalizing and externalizing mental illness symptoms predicted higher intrapersonal motives regarding NSSI. These results can be easily incorporated into the Experiential Avoidance Model (EAM; [59]). Deficiencies in emotion regulation, such as poorer distress tolerance and mental health status, may generate intense emotions which could activate NSSI acts to avoid overwhelming aversive emotions [59].

A pattern somewhat opposite that observed with intrapersonal functions emerged when examining interpersonal functionality. Lower loneliness, lower internalizing symptoms, and younger age were associated with higher interpersonal motivation for self-injury; however, the explained variance was relatively low. These results indicate that younger adolescents with fewer mood and anxiety symptoms, but with a wider social circle, could be more responsive and vulnerable to engaging in self-injury because of interpersonal than intrapersonal reasons. Further research could potentially examine peers of adolescents and how their reactions play a role in the adolescent’s motivation for self-injury.

### Limitations

Our study examined a generally healthy population. Although we surveyed mental health symptoms (i.e., internalizing and externalizing symptoms), but for ethical reasons, we did not ask specific questions about former or current psychological disorders, nor did we inquire regarding pharmacotherapy or psychological treatment. We also neglected these topics because self-reported psychiatric history is less reliable and we had no opportunity to conduct more reliable clinical diagnostic interviews. Therefore, it is possible that our sample contains participants who have had different types of mental illness syndromes which may have influenced our results.

Another main limitation was the relatively low sample size of male adolescents. In further studies, it would be important to increase and equalize the proportion of male participants. Doing so might provide more data analytic options that could be especially helpful in reconciling the somewhat contradictory results about gender differences in pure NSSI groups [25]. Moreover, multiple analyses had been conducted with complex models in a relatively small sample, and therefore caution should be urged regarding interpretations. Type I error was partly compensated for by using a Bonferroni correction for some analyses based on relatively low sample sizes. Furthermore, there is evidence that individuals engaging in more chronic and severe NSSI differ from those who engage in infrequent NSSI [60]. However, low subsample sizes in our study inhibited running ESEM analyses separately in the two severity (occasional and repetititve self-injury) groups. Further work on larger samples may reveal that, in the two severity groups, the factor structure of the NSSI motives are different.

Another limitation of our research is the cross-sectional study design, therefore, we could not provide causal relationships in the explanatory analyses. Furthermore, the study was based on self-report measures completed in classrooms. Although trained investigators supervised and helped the process of collective testings and no teaching staff were present in the classes, there was a risk of less than honest responses because of the sensitive topic and social desirability.

Finally, we utilized the short form of the ISAS Part II. There is a risk of construct and statistical underrepresentation because only two indicators were used for factors, which is less suitable for determining a latent variable. However, it should be noted that the original long form of the ISAS Part II uses three indicators per subscales.

### Conclusion

Based on ESEM approach, this study can contribute to the factor structure analyses of the ISAS part II. In addition to confirmation of the two-factor structure (i.e., intra- and interpersonal factors) of the questionnaire, we also pointed out that this structure varies across gender. In the girls’ sample, we detected robust associations between intrapersonal functionality of NSSI and poorer mental health and emotion regulation aspects, as well as more frequent NSSI.

## Supplementary Information

**Additional file 1.**

**Additional file 2.**

## Data Availability

The dataset used and analyzed during the current study is available from the corresponding author on reasonable request.

## Abbreviations

AFQ-Y8: Avoidance and Fusion Questionnaire for Youth
CASE: Child & Adolescent Self-Harm in Europe Study
CFA: confirmatory factor analyses
CFI: Comparative Fit Index
EAM: Experiential Avoidance Model
EFA: exploratory factor analysis
ESEM: exploratory structural equation modeling
FFM: Four Function Model
ISAS: Inventory of Statements About Self-Injury
MIMIC: Multiple Indicator Multiple Causes
NSSI: nonsuicidal self-injury
NSSID: nonsuicidal self-injury disorder
RMSEA: Root Mean Squared Error of Approximation
SCRS: Self-Critical Rumination Scale
SDQ: Strength and Difficulties Questionnaire
TLI: Tucker-Lewis Index
WHO: World Health Organization
WLSMV: Weighted Least Squares Mean and Variance
