# Supplementary material for: Functions of nonsuicidal self-injury in a Hungarian community adolescent sample: a psychometric investigation
Source: BMC Psychiatry. 2021 Dec 9;21:618. doi: 10.1186/s12888-021-03613-4 (PMC8662905; doi:10.1186/s12888-021-03613-4)
Supplement: Supplementary file 1 — Additional file 1. [file 12888_2021_3613_MOESM1_ESM.docx]

**Supplementary Table 1**

*Means, standard deviations of NSSI functions, and t-statistics and effect sizes by gender among adolescents who have engaged in NSSI*

|  | Total (N=351)  M (SD) | Boys  (n=98; 27.92%)  M (SD) | Girls (n=253; 72.08%)  M (SD) | Test statistic  t (p) | Effect size  (Cohen-d) |
| --- | --- | --- | --- | --- | --- |
| *NSSI functions* |  |  |  |  |  |
| Affect regulation | 2.19 (1.51) | 1.77 (1.42) | 2.53 (1.51) | **3.32 (<0.001)** | 0.51 |
| Self-punishment | 1.45 (1.45) | 1.02 (1.12) | 1.61 (1.52) | **3.48 (<0.001)** | 0.42 |
| Anti-dissociation | 1.28 (1.42) | 0.90 (1.20) | 1.43 (1.47) | **3.20 (0.002)** | 0.38 |
| Anti-suicide | 1.85 (1.29) | 1.89 (1.41) | 1.83 (1.24) | 0.35 (0.726) | 0.05 |
| Marking distress | 0.79 (1.09) | 0.67 (1.04) | 0.83 (1.11) | 1.21 (0.228) | 0.15 |
| Interpersonal boundaries | 0.82 (1.12) | 0.70 (0.97) | 0.87 (1.17) | 1.24 (0.215) | 0.15 |
| Self-care | 0.58 (0.99) | 0.64 (1.05) | 0.55 (0.97) | 0.76 (0.447) | 0.09 |
| Sensation seeking | 0.49 (0.94) | 0.76 (1.16) | 0.38 (0.82) | **3.37 (<0.001)** | 0.41 |
| Peer bonding | 0.86 (1.23) | 1.02 (1.39) | 0.80 (1.16) | 1.52 (0.129) | 0.18 |
| Interpersonal influence | 0.81 (1.10) | 0.80 (1.07) | 0.81 (1.11) | 0.14 (0.889) | 0.01 |
| Toughness | 0.97 (1.19) | 1.11 (1.23) | 0.92 (1.16) | 1.33 (0.184) | 0.16 |
| Autonomy | 0.63 (1.00) | 0.70 (1.05) | 0.60 (0.98) | 0.87 (0.386) | 0.10 |
| Revenge | 0.34 (0.78) | 0.46 (0.90) | 0.30 (0.72) | 1.77 (0.078) | 0.21 |

*Note*. Gender-based comparisons related to NSSI functions were performed with independent sample t-tests. Test statistic values presented with bold figures are significant at least p < 0.05 level. Based on Bonferroni correction (p = 0.0038) all significant values remain significant.
