# Supplementary material for: Functions of nonsuicidal self-injury in a Hungarian community adolescent sample: a psychometric investigation
Source: BMC Psychiatry. 2021 Dec 9;21:618. doi: 10.1186/s12888-021-03613-4 (PMC8662905; doi:10.1186/s12888-021-03613-4)
Supplement: Supplementary file 2 — Additional file 2. [file 12888_2021_3613_MOESM2_ESM.docx]

**Supplementary Table 2**

*Zero-order correlations of the variables among girls who engage(d) in self-harm*

|  | 1. | 2. | 3. | 4. | 5. | 6. | 7. | 8. | 9. | 10. | 11. | 12. | 13. | 14. | 15. | 16. | 17. | 18. | 19. | 20. |
| --- | --- | --- | --- | --- | --- | --- | --- | --- | --- | --- | --- | --- | --- | --- | --- | --- | --- | --- | --- | --- |
| 1. Age | - | -0.01 | 0.002 | 0.08 | 0.03 | 0.10 | -0.04 | 0.10 | 0.01 | 0.02 | -0.01 | 0.03 | -0.02 | -0.02 | 0.08 | *-0.13* | *-0.13* | 0.002 | -0.11 | *-0.17* |
| 2. Loneliness# |  | - | ***-0.43*** | ***-0.53*** | ***-0.56*** | ***-0.23*** | *0.15* | ***-0.29*** | ***-0.41*** | ***-0.32*** | *0.15* | *-0.17* | *-0.17* | 0.12 | 0.06 | ***0.25*** | 0.05 | -0.06 | -0.07 | 0.02 |
| 3. AFQ-Y8 |  |  | - | ***0.62*** | ***0.52*** | ***0.35*** | *-0.15* | ***0.33*** | ***0.43*** | ***0.35*** | -0.08 | *0.21* | ***0.23*** | -0.002 | 0.01 | -0.11 | 0.03 | 0.09 | 0.04 | *0.13* |
| 4. SCRS |  |  |  | - | ***0.65*** | ***0.24*** | *-0.12* | ***0.32*** | ***0.52*** | ***0.28*** | *-0.16* | *0.21* | ***0.23*** | *-0.16* | -0.11 | -0.11 | 0.003 | 0.06 | 0.06 | 0.04 |
| 5. SDQ internalizing |  |  |  |  | - | ***0.26*** | *-0.21* | ***0.26*** | ***0.37*** | ***0.29*** | *-0.13* | *0.16* | *0.21* | -0.09 | -0.04 | *-0.15* | 0.003 | 0.05 | 0.02 | 0.08 |
| 6. SDQ externalizing |  |  |  |  |  | - | *-0.21* | *0.16* | *0.18* | ***0.24*** | 0.06 | *0.13* | *0.15* | 0.05 | *0.21* | -0.07 | 0.10 | *0.17* | 0.09 | *0.15* |
| 7. SDQ prosocial |  |  |  |  |  |  | - | -0.08 | 0.03 | -0.03 | 0.08 | -0.01 | -0.02 | 0.03 | -0.06 | 0.11 | 0.06 | -0.03 | 0.04 | -0.07 |
| 8. Affect regulation* |  |  |  |  |  |  |  | - | ***0.51*** | ***0.49*** | -0.01 | ***0.40*** | ***0.26*** | 0.05 | 0.09 | *-0.18* | 0.02 | *0.13* | 0.01 | 0.09 |
| 9. Self-punishment* |  |  |  |  |  |  |  |  | - | ***0.46*** | -0.11 | ***0.35*** | ***0.34*** | -0.03 | 0.11 | *-0.14* | 0.02 | *0.20* | *0.13* | 0.09 |
| 10. Anti-dissociation* |  |  |  |  |  |  |  |  |  | - | -0.04 | ***0.42*** | ***0.34*** | 0.03 | *0.20* | *-0.17* | 0.04 | ***0.28*** | *0.16* | 0.12 |
| 11. Anti-suicide* |  |  |  |  |  |  |  |  |  |  | - | 0.10 | *0.15* | ***0.29*** | *0.14* | ***0.30*** | ***0.29*** | *0.21* | ***0.35*** | *0.14* |
| 12. Marking distress* |  |  |  |  |  |  |  |  |  |  |  | - | ***0.30*** | 0.11 | 0.05 | 0.04 | ***0.38*** | ***0.32*** | ***0.24*** | ***0.28*** |
| 13. Interpers. boundaries* |  |  |  |  |  |  |  |  |  |  |  |  | - | ***0.27*** | ***0.27*** | 0.08 | *0.15* | ***0.23*** | ***0.36*** | *0.21* |
| 14. Self-care* |  |  |  |  |  |  |  |  |  |  |  |  |  | - | ***0.24*** | ***0.38*** | ***0.37*** | *0.22* | ***0.29*** | ***0.23*** |
| 15. Sensation seeking* |  |  |  |  |  |  |  |  |  |  |  |  |  |  | - | 0.06 | -0.01 | ***0.42*** | ***0.26*** | *0.21* |
| 16. Peer bonding* |  |  |  |  |  |  |  |  |  |  |  |  |  |  |  | - | ***0.50*** | 0.12 | ***0.36*** | *0.18* |
| 17. Interpers. influence* |  |  |  |  |  |  |  |  |  |  |  |  |  |  |  |  | - | *0.18* | ***0.28*** | ***0.26*** |
| 18. Toughness* |  |  |  |  |  |  |  |  |  |  |  |  |  |  |  |  |  | - | ***0.43*** | *0.17* |
| 19. Autonomy* |  |  |  |  |  |  |  |  |  |  |  |  |  |  |  |  |  |  | - | ***0.33*** |
| 20. Revenge* |  |  |  |  |  |  |  |  |  |  |  |  |  |  |  |  |  |  |  | - |

*Note.* N=251. #: Reversed item. Correlation coefficients presented with italicized figures are significant at least p < 0.05 level. Correlation coefficients presented with bold italicized figures are significant based on Bonferroni correction (p = 0.0025). AFQ-Y8=Avoidance and Fusion Questionnaire for Youth. SCRS=Self-Critical Rumination Scale. SDQ=Strength and Difficulties Questionnaire. SDQ internalizing=Strength and Difficulties Questionnaire internalizing symptoms. SDQ externalizing=Strength and Difficulties Questionnaire externalizing symptoms. SDQ prosocial=Strength and Difficulties Questionnaire prosocial behavior.

* Self-harm motives according to the Inventory of Statements About Self-Injury (ISAS).
